# The role of plants in the formation of species-specific features in grass flies (Diptera, Chloropidae, Meromyza)

**DOI:** 10.3897/BDJ.9.e78017

**Published:** 2021-12-29

**Authors:** Tatiana A. Triseleva, Varos G. Petrosyan, Aleksandra A. Yatsuk, Andrey F. Safonkin

**Affiliations:** 1 A.N. Severtsov Institute of Ecology and Evolution, Moscow, Russia A.N. Severtsov Institute of Ecology and Evolution Moscow Russia

**Keywords:** morphometric, mtDNA CO1, postgonites, Poaceae, co-evolution insect-plant

## Abstract

In the current manuscript, we present the results of comparative analysis of seven species of *Meromyza* flies in the “*variegata*” cluster and of the evolutionary close species *M.inornata*, based the following criteria: 1) 14 external key features; 2) shape and area of the anterior processes of postgonites; 3) mtDNA CO1 region and 4) host plant diversity data. We could demonstrate the primary role of host plants in species formation inside genus *Meromyza* and calculated the timing of the divergence of *M.inornata* and the species of “*variegata*” cluster. Based on our estimates of evolution rate for mtDNA CO1 gene, we could conclude that that divergence of herbs happened before the speciation of grass flies *Meromyza*. *Meromyza* species, close to the ancestral species of the cluster, are adapted to the wide range of host plants. We revealed the most informative variables h1, S and Plant analysing data with the following statistical methods: linear discriminant analysis - LDA, regularised discriminant analysis - RDA, flexible discriminant analysis – FDA and probabilistic neural network - PNN. The highest classification accuracy was achieved using PNN (99%) and the lowest when using LDA (95.8%). When the Plant trait was excluded, the classification accuracy decreased by 14%. We revealed the significant trends in size change of the anterior process of the postgonite amongst studies species. This morphological structure is an element of male reproductive apparatus critical for the restriction of interspecies mating. We determined three branches of speciation in the “*variegata*” cluster and five trends in the evolution of this cluster, based on the external morphological features. We showed that *M.variegata* and especially *M.mosquensis*, the species closest to the ancestral haplotype, have the largest number of features typical of those of *M.inornata.* Based on the external features and the area of the anterior process of the postgonite, we reconstructed the phylogenetic position of *M.elbergi* in the cluster. In accordance with the obtained outcomes, we could conclude that the distribution, species diversity and the adaptation of the grass flies to narrow oligophagy were directly connected to host plant diversity. The adaptation to different host plants could be the main factor in divergence of grass flies and their evolution started later than the diversification in the Pooideae subfamily of grasses.

## Introduction

Studies of the mechanisms and factors contributing to species biodiversity are an important part of modern research in zoology (Chesson 2000, Vorobjeva and Striganova 2005, Parmesan 2006, Gaujour et al. 2012, Jeltsch et al. 2013). Modern approaches to phylogenetic reconstructions are based on interaction of cladistics, numerical and genetic phyletics (Scotland et al. 2003, Pavlinov 2003, Pavlinov 2005, Patwardhan et al. 2014). In classical phylogenetics, construction of a cladogram represents the initial stage of phylogenetic studies where reconstruction of an evolutionary scenario requires a set of additional data (Pavlinov 2005). In contrast, the creation of a cladogram in modern phylogenetics is the final stage of phylogenesis reconstruction.

Grass flies of the genus *Meromyza* (Diptera, Chloropidae, *Meromyza* Meigen, 1830) represent a perfect model for employment of both modern and classical approaches to phylogenetic reconstructions. To date, *Meromyza* includes more than 90 species, distributed throughout the Northern Hemisphere. The identification key of this genus was developed, based on a set of external morphological features and specific features of male genital apparatus (Narchuk and Fedoseeva 2010).

The results of genetic analysis made it possible to divide the genus into eight clusters (Safonkin et al. 2016) which triggered the question about the factors contributing to diversification of this genus. The change of host plant is important in speciation of phytophages (Forbes et al. 2017). Assuming that evolution of host plants can be an important factor, we looked closely into a list of host plants for one third of the species of *Meromyza* flies (Panteleeva 1989, Nartshuk and Fedoseeva 2011). Additionally, the genetic analysis has allowed us to trace the formation of species groups (clusters) within the genus, as well as the relative time of speciation within these groups and the degree of species nutritional adaptation (Safonkin et al. 2020a). However, no data are available about the development of specific features, in particular, for species during the evolution of genus or the role of host plant. In flies of genus *Meromyza*, all host plants, the area, the relative time of speciation, the degree of relationship between species, based on the postgonite structure and the mtDNA COI locus are currently known only for a representative sample of species from the “*variegata*” cluster.

Species of the “*variegata*” cluster are widespread throughout Europe (Safonkin et al. 2020b). These species show striking similarities despite wide variability of external features, but for species-specific male genital apparatus (Safonkin et al. 2020b). Some species within the cluster have a broad range of host plants, but other species are specialised in limited host plant groups (Panteleeva 1989, Nartshuk and Fedoseeva 2011).

In the current manuscript, we show the results from our study of the distribution of various external key features and structural features of male genital apparatus amongst the species of “*variegata*” cluster. We also present here the results of comparative analysis of seven species of *Meromyza* flies in the “*variegata*” cluster and evolutionary close species *M.inornata* Becker, 1910, based on external key features, shape and area of the anterior processes of postgonites, mtDNA CO1 region and host plant diversity data. We demonstrate the important role of host plants in species formation inside genus *Meromyza*.

## Material and methods

The comparative analysis was based on the original descriptions of *M.bohemica* Fedoseeva, 1962, *M.elbergi* Fedoseeva, 1979, *M.femorata* Macquart, 1835, *M.laeta* Meigeni, 1838, *M.mosquensis* Fedoseeva 1960, *M.rufa* Fedoseeva. 1962, *M.variegata* Meigeni, 1830 and*M.inornata* (Becker 1910, Fedoseeva 1960, Fedoseeva 1962, Hubicka 1970, Fedoseeva 1979) and results of our morphometric study of specimens from the collection of the Severtsov Institute of Ecology and Evolution (Moscow, Russia), partially presented earlier (Safonkin et al. 2020b).

**Genetic analysis.**

We analysed the relationship and time of species divergence, based on the nucleotide sequences of mtDNA CO1 locus previously deposited by us in GenBank. A phylogenetic tree was constructed via the Bayesian approach using the BEAST v.1.10.4 software package with default parameters, except for the Tree Prior parameter, for which the G. Yule speciation model was chosen. The numbers in the nodes indicate the replacement for the site for 1 million years (Triseleva et al. 2014, Safonkin et al. 2016, Safonkin et al. 2020b).

**Methods for species differentiation, based on comparative analysis of external features and male genitals.**

For comparative analysis in 66 males and 58 females of eight species of grass flies, we have selected the most distinct features (out of 35 analysed): eight quantitative features: h1 (ratio of the gena height to the height of the 3rd antennal segment), h2 (ratio of the length of triangle to the length of head), h3 (ratio of the height of ocellar triangle to the base of triangle), h4 (ratio of the width of hind femur to the width of hind tibia), L (length of the body without abdomen), L1 (ratio of the length of head to the length of mesonotum), L2 (ratio of mesonotum to scutellum), S (area of anterior process of the postgonite) and six qualitative traits (Table 1). Additionally, we analysed three ecological characters: habitat, biotope and species of host plants.

For comparative analysis of the shape of anterior process of the postgonites, we used our previous data (Yatsuk and Safonkin 2018).

**The analysis of host plants.**

The species of host plants are taken from the work of Safonkin et al. (2020b). To time the origin of grasses, we used data from the work of Pimentel et al. (2017).

**Statistical methods for species differentiation, based on quantitative and qualitative traits.**

Evaluation of the separating ability of the selected quantitative (8) and qualitative (1) traits for the differentiation of 96 individuals, belonging to seven species of grass flies, was carried out using two types of methods. The first type includes various forms of discriminant analysis (DA) (Friedman 1989, Petrosyan 2014, Shitikov and Mastitsky 2017) (linear discriminant analysis - LDA and regularised discriminant analysis - RDA, flexible discriminant analysis - FDA). The second type includes methods based on artificial neural networks, in particular, probabilistic neural network (PNN) (Specht 1990, Petrosyan 2014, Mohebali et al. 2020). The use of different methods of DA is due to the fact that these methods have different requirements for the dataset, in particular, the LDA method assumes that the data distribution in each class is normal and that the intragroup variance and correlation matrices are equal. Another DA method, RDA, builds a species differentiation rule, based on information features by regularising group covariance matrices, allowing a more reliable model to be created taking into account the multicollinearity of the data. This technique is usually useful for large multidimensional data containing highly correlated predictors. The third modification method of DA is FDA, which is a flexible extension to LDA that uses non-linear combinations of predictors, such as splines. The FDA is known to be useful for modelling multidimensional abnormal and non-linear relationships between variables in each group, resulting in a more accurate classification.

It is important to note that DA methods work only with quantitative traits. In our case, there is one quality feature (the number of host plant species). For this reason, PNN is used to assess the informative nature of this trait for the separation of species.

To determine the set of linear discriminant functions (LDF) separating species, we used a stepwise discriminant analysis procedure with the threshold value of the inclusion of variables F = 4 (Petrosyan 2014). A quantitative assessment of the distinguished discriminant functions was carried out using a set of parameters, including eigenvectors, coefficients of canonical correlations for discriminant functions, Wilkes statistics, С hi-square statistics, as well as P-values of testing hypotheses for the significance of the separation of species (Petrosyan 2014). After the selection of informative features, the quality of the LDA model was assessed using a leave-one-out CV-procedures (Shitikov and Mastitsky 2017). For other forms of discriminant analysis in FDA and RDA, we also used a cross-check procedure for the final assessment of the quality of the models (Shitikov and Mastitsky 2017).

In the present study we use PNN, which has four layers: input, pattern, summation and output (Specht 1990, Petrosyan 2014, Mohebali et al. 2020). The task of the input layer is to distribute the input layer data for the pattern layer. The number of input signals is equal to the number of variables that define the samples (individuals). The pattern layer has one element for each sample (individual) from the training dataset. The input layer and the sample layer form a fully-connected structure. The output layer consists of seven neurons that determine whether an individual belongs to one of the seven species based on voting, taking into account the signals received from the summation layer. Effective methods for constructing PNNs are presented in the literature (Specht 1990, Petrosyan 2014, Mohebali et al. 2020). In this PNN, information moves in only one direction - forward from the input nodes (neurons) through the pattern layer to the output layer (neurons). As input variables of the network, we used all the quantitative variables that were used in the LDA, FDA and RDA methods and, additionally, we added the variable plant. The assessment of the classification accuracy was determined by averaging the accuracy of a 24-fold repetition of the network models. At each run, 92 individuals were randomly used as training and four individuals for testing (Petrosyan 2014). The small amount of selection of individuals to test the accuracy of the PNN-models is due to the fact that two species *M.rufa* and *M.laeta* are represented by a limited number of some features, three and four, respectively.

All assessments with the method of discriminant analysis were carried out in the RStudio v. 1.4.1106 using basic, special R-packages (MASS, klaR, mda, tidyverse, caret, dplyr, FactoMineR) and additional programmes in the R language. Probabilistic neural networks were created using the Biosystem office (Petrosyan 2014).

## Results

**Assessment of species diversity, based on genetic analysis**

Сlustering of *Meromyza* species, based on the mtDNA CO1 gene, revealed two close clusters. The “*variegata*” cluster included six species and the “*inornata*” cluster included only the single species because, currently, we have no other allied species.

**Figure 1.**
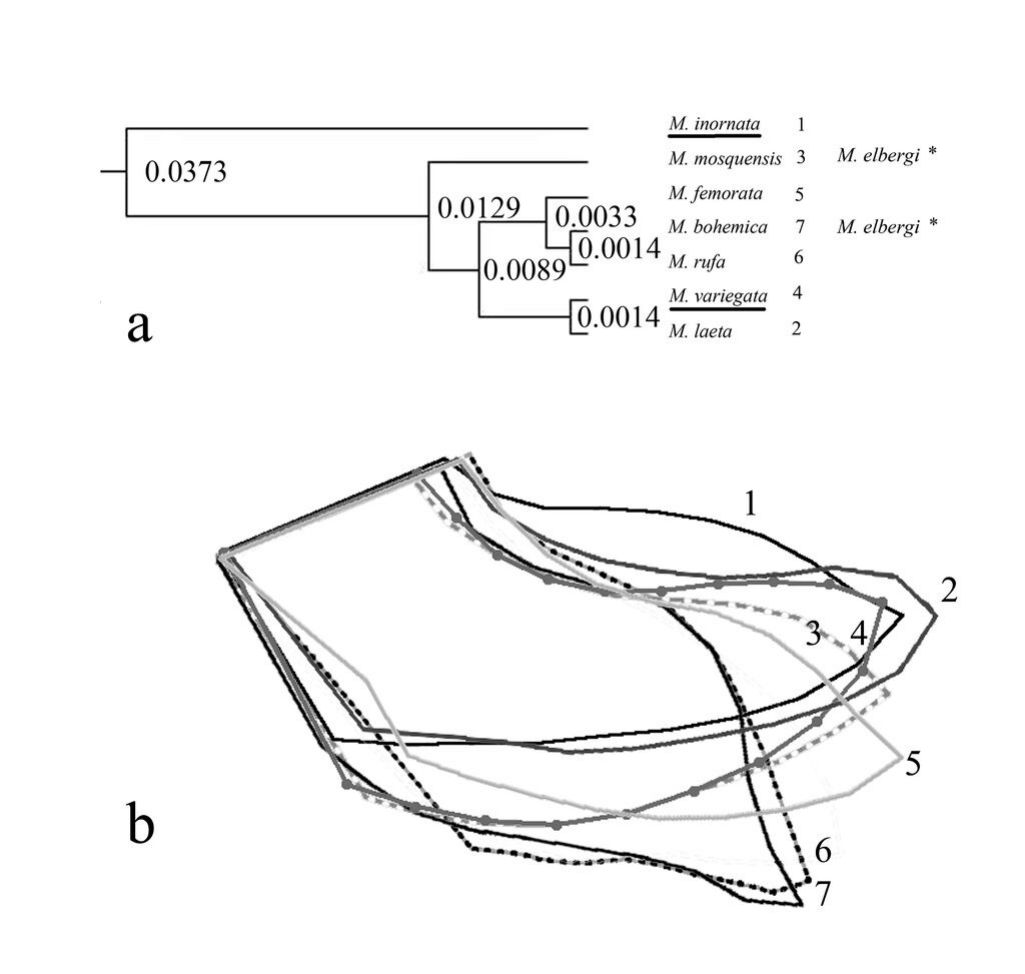
A phylogenetic tree of species of the “*variegata*” and “*inornata*” clusters and postgonites shape: **a** phylogenetic tree, based on the mtDNA CO1, constructed in the programme BEAST v.1.10.4. (partially from fig. 1 by Safonkin et al. 2020a). The numbers in the nodes indicate the replacement for the site for 1 million years. Underlined are the species that gave the name to the clusters. Vertical lines – evolutionary lineages of the “*variegata*” cluster. * – possible position of *M.elbergi* in the “*variegata*” cluster. **b** shape of anterior process of the postgonites of *M.inornata* (1), *M.laeta* (2), *M.mosquensis* (3), *M.variegata* (4), *M.femorata* (5), *M.rufa* (6) and *M.bohemica* (7).

There are three branches in the “*variegata*” cluster: 1). *M.variegata* and *M.laeta*, which are closer to the hypothetical ancestor of the cluster; 2). *M.mosquensis*; and 3). the more recent species *M.femorata, M.rufa* and *M.bohemica* (Fig. 1). The timing of the divergence of *M.inornata* and the species of the “variegata” cluster is shown in Table 2. The time was calculated according to the estimated time of the origin of the genus *Meromyza* from 15.8 to 4.02 million years (Safonkin et al. 2020a).

**Assessment of species diversity, based on external features and male genitals.**

Based on the combination of morphological features, we include the seventh species *M.elbergi* into the “*variegata*” cluster. We can reveal five trends in the evolution of the “*variegata*” cluster, based on the external key features (Table 1):

1. Pale colour of the mid-stripe and occipital spot, but bright occipital strips (except for *M.mosquensis* and *M.elbergi*);
2. Black setae on the lower surface of gena in *M.bohemica*, sometimes, in *M.elbergi*, *M.femorata*, *M.variegata*;
3. *M.inornata, M.femorata* and *M.elbergi* are close by the height of gena. The remaining species of the cluster are characterised by a decrease in the height of the gena. Closely related species *M.rufa - M.bohemica* are strikingly different;
4. Based on the ratio of the height and width of the ocellar triangle, closely related species are separated by head capsule stretch (*M.variegata* – *M.laeta*, *M.mosquensis* – *M.elbergi*, *M.rufa* – *M.bohemica*);
5. The considerable thickening of the hind femur in *M.femorata*, *M.variegata* and *M.rufa.* The other key features are almost non-distinguishable. The area of the anterior process of postgonite significantly differs in most species of the “*variegata*” cluster.

The area of the anterior process of the postgonite is maximal in species close to the common hypothetical ancestor (*M.inornata*, *M.variegata*) and decreases in younger species (*M.variegata* - *M.laeta*, *M.mosquensis* - *M.elbergi*, *M.femorata* - *M.rufa*, *M.bohemica*) (Table 1). The shape of the anterior process of the postgonite is similar in *M.variegata* and *M.inornata*, but different considering the degree of curvature of the upper and lower contours. In *M.laeta*, the shape of the anterior process of the postgonite is acuminate. In other species, the anterior process of the postgonite is curved forward (Fig. 1b).

The number of grasses suitable for development of flies is maximum in *M.variegata* (six plants) and *M.mosquensis* (six or seven plants), fewer in *M.laeta, M.femorata* and *M.rufa* (three plants for each fly species) and one host plant in *M.bohemica* (Table 3).

**Discriminant analysis results**

Using a stepwise selection algorithm, it was determined that six variables (S, h1, h2, L1, L and h4) were significant predictors of species (Tables 4, 5). The order of these variables is given by the importance of their inclusion in the LDA model. The stepwise selection algorithm in LDA showed that four discriminating functions have P-values less than 0.05, i.e. are statistically significant at the 95.0% confidence level (Table 5). In our case, although four functions are statistically significant, nevertheless, the first two functions account for the overwhelming majority of the separation of species, i.e. Wilkes' statistics, the relative percentage of discrimination and the canonical correlation coefficient for the first two functions are 0.003, 85.79%, 0.98 and 0.82, 9.26%, 0.86, respectively (Table 5). For the other two functions, the relative percentages of discrimination are 3.4% and 1.23%, respectively. Canonical correlation coefficients indicate that each subsequent discriminant function contributes less to discrimination than the previous one (Table 4). Unlike the first discriminant function, which has a canonical correlation coefficient of 0.98, for the fifth, this coefficient is 0.28. The classification accuracy using the four LDA functions is presented in Table 6.

**Figure 2.**
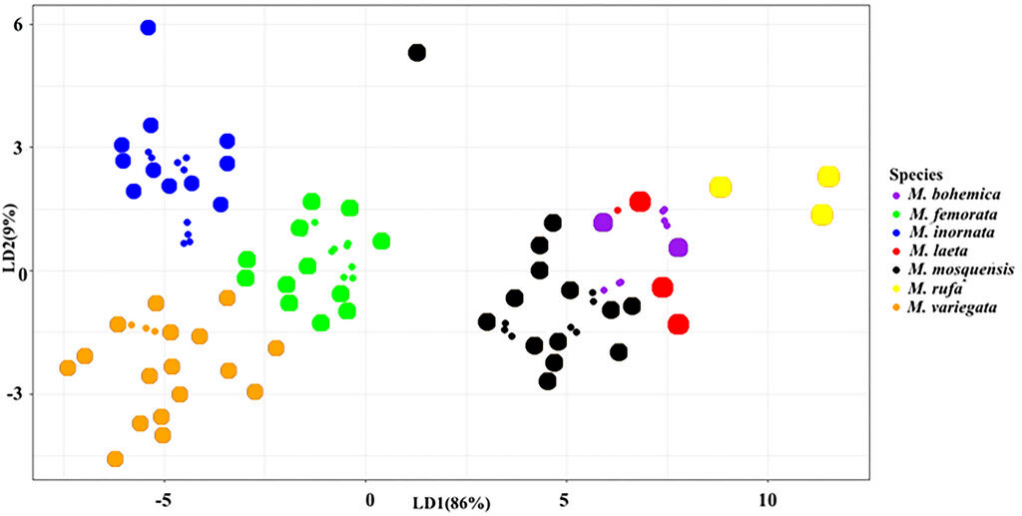
Species projection on the first (LD1) and second (LD2) discriminant functions.

The application of the leave-one-out CV procedure showed that the selected LDA discriminant functions allow species classification with an accuracy of 95.83% (Table 6). Species projection onto the first linear discriminants LD1 and LD2 is shown in Fig. 2.

**Figure 3.**
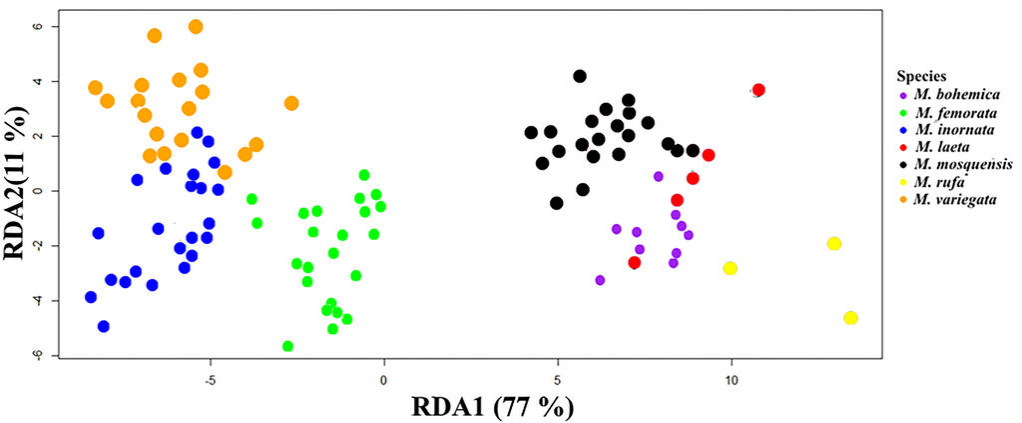
Regularised discriminant analysis projection on the first (RDA1) and second (RDA2) canonical axes.

The use of other methods of DA in the form of RDA and FDA using six traits (S, h1, h2, L1, L and h4) showed that the classification accuracy of these methods does not improve significantly. In the RDA methods of classification, the numbers of correct and incorrect classification of individuals are 94 and 2, respectively (Table 7), i.e. the classification accuracy is 97.92%. Regularised discriminant analysis projection on the first (RDA1) and second (RDA2) canonical axes is shown in Fig. 3.

**Figure 4.**
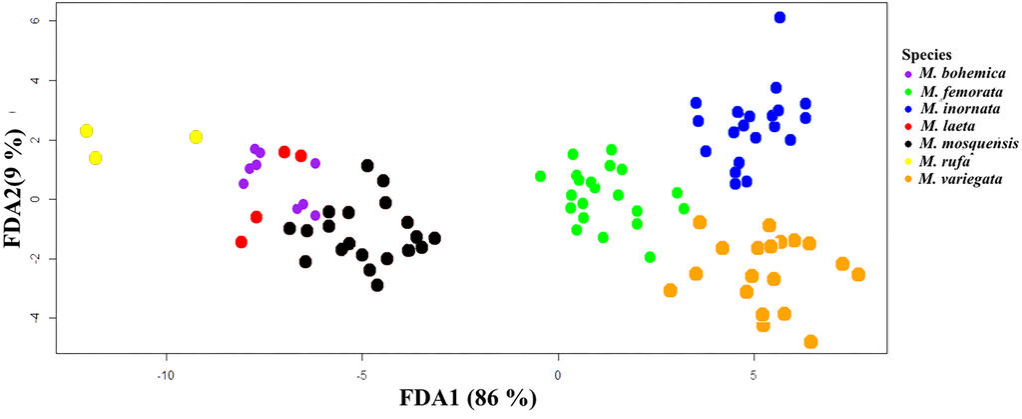
Flexible discriminant analysis projection of seven species on the first (FDA1) and second (FDA2) canonical axes.

The highest classification accuracy is achieved using the FDA method. In the FDA method, the numbers of correct and incorrect classification of individuals are 95 and 1, respectively (Table 8), i.e. the classification accuracy is 98.96%. Flexible discriminant analysis projection of seven species on the first (FDA1) and second (FDA2) canonical axes is shown in Fig. 4.

**The results of using a PNN.**

**Figure 5.**
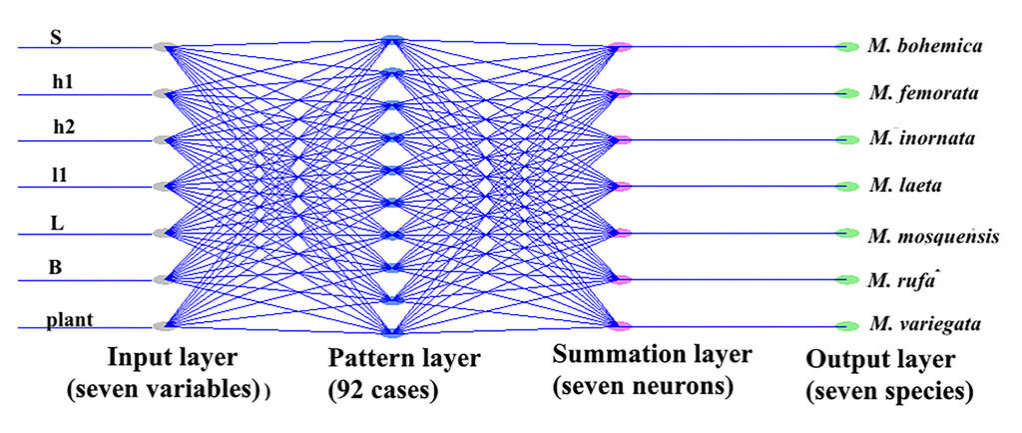
General architecture of a PNN.

The general architecture of a PNN, which was used to differentiate seven species, based on quantitative (6) and qualitative (1) features, is shown in Fig. 5. Application of PNN to the seven species of flies showed that the use of a qualitative variable plant further improves the classification results.

**Figure 6.**
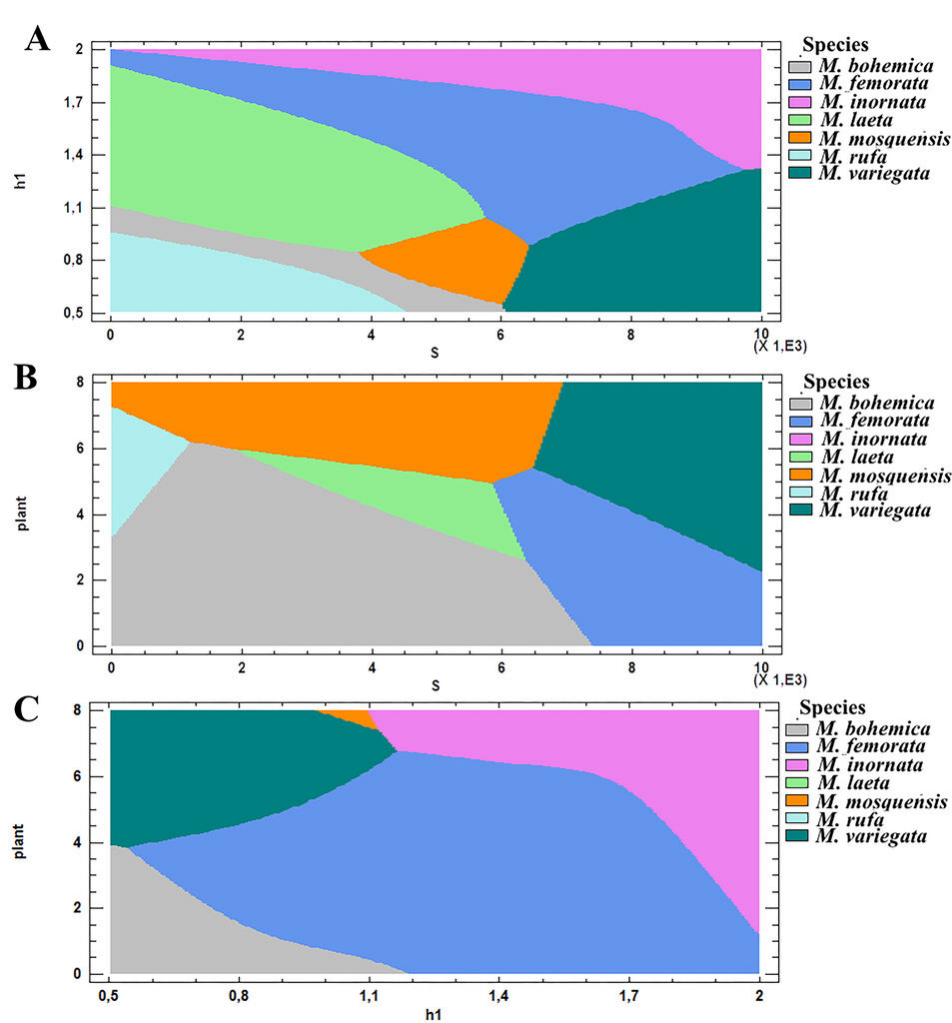
Areas of change in the values of the most important traits h1, S and Plant for individuals of seven species, determined using a PNN with fixed (average) values of the remaining variables.

**Figure 7.**
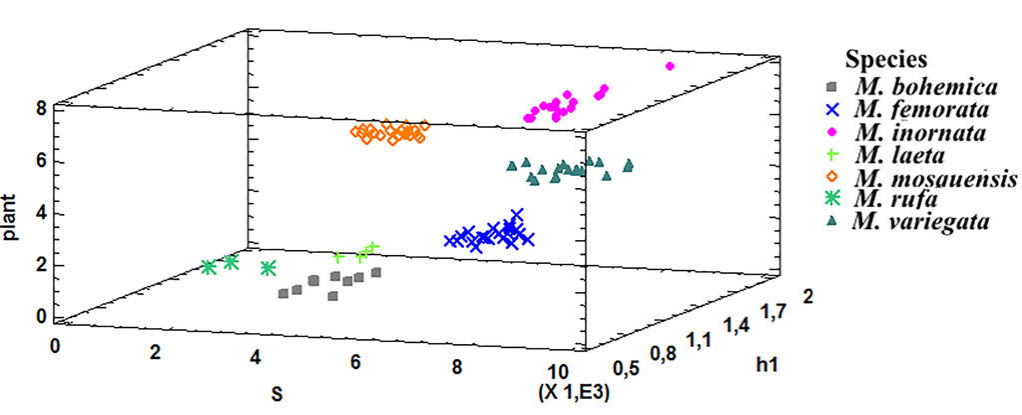
Three-dimensional scatter plot of seven species individuals in the space of three informative features (S, h1 and Plant).

The conducted 24 realisations of PNN models showed that the classification accuracy on the training samples is 98.5% (± 0.3) and on the verification (testing) samples is 99% (± 1.1). To visualise the species differentiation, as an example, Fig. 6 shows the areas of change in the values of traits of individuals of seven species, which are used by PNN to differentiate individuals. These diagrams represent the areas of change in the values of features that characterise the most informative variables h1, S and Plant. Fig. 6A shows that each species in the plane S and h1 has its own characteristic area, determined by the ranges of variation of the variables S and h1. It is important to note that the diagram in Figure 6A is divided into seven parts, i.e. each species is characterised by a certain area of definition of the variables h1 and S. However, this statement is not fulfilled in terms of informative features of Plant and S (Fig. 6B). In the plane of these variables, the areas for six species, with the exception of *M.inornata*, are clearly distinguished. This diagram shows that the area of variation for Plant and S traits for *M.inornata* overlaps with that for *M.variegata* (see Fig. 7).

In the plane of informative features for Plant and h1, the areas of variation of these variables are presented for five species (Fig. 6C), with the exception of *M.laeta* and *M.rufa*. This diagram shows that, with the average values of the remaining variables, the area of variation for Plant and h1 traits for *M.bohemica* overlaps with that for *M.laeta* and *M.rufa* (Figs 6, 7), which indicates close values of Plant and h1.

To check the differentiating importance of the Plant trait in PNN, we built another network with six traits without Plant trait. Assessments, based on 24 realisations of PNN models using six features, showed that the classification accuracy on training samples is 84.8% (± 0.4) and, on testing samples, is 90% (± 2.7). When this trait is excluded, the classification accuracy decreased by 14%.

Typical errors in the classification of individuals of the species when using different methods of DA are presented in Tables 6-8. Typical errors in PNN are associated with the assignment of one *M.laeta* to the *M.bohemica* species and vice versa, as well as the *M.inornata* individual to another *M.variegata* species.

## Discussion

Based on the molecular clock of insect mtDNA CO1, the divergence rate is about 1.5 - 4% per one million years (from 0.0075 to 0.012 substitutions per site (Rowan and Hunt 1991, Brower 1994, Jamnongluk et al. 2003) with an average value of 2.75% (Papadopoulou et al. 2010). Based on the known mutation rate of the CO1 gene of mtDNA in *Drosophila* Fallén, 1823 we could extrapolate the time of origin of the common ancestor of *Meromyza* at 15.8 - 4.02 million years (Safonkin et al. 2020a). We estimate that the rate of gene evolution of *Meromyza* flies is more likely closer to the lower value. Thus, the maximum time of divergence of the ancestors of *M.inornata* and the “*variegata*” cluster and the speciation of *M.variegata* can be estimated at 6.58 million years and the period of divergence of *M.mosquensis* “lineage” - 2.27, *M.femorata* “lineage” - 1.57 million years, respectively.

Taxonomic divergence in Pooideae (the main host plants of *Meromyza* flies) began in the middle of the Eocene - the beginning of the Oligocene and resulted in ecological dominance in the Northern Hemisphere at present (Pimentel et al. 2017). Grass diversity increased from the Middle to Late Miocene, during which the formation of open grass biomes of cold and warm climates also took place everywhere on earth (Strömberg 2005, Spriggs et al. 2014). Grass-dominated biomes of Western Eurasia formed 21–20 million years ago (Strömberg 2011). Based on our estimates of evolution rate for the above gene, we can conclude that divergence of herbs was before the speciation of grass flies *Meromyza*. For example, the speciation time of host grass *Loliumperenne* L. (Table 3) exceeds the speciation time of the youngest species of the cluster, *M.bohemica* by ten times.

The adaptation to phytophagy in some dipterans was probably linked to climatic deterioration in the Neogene and the formation of new trophic connections. Tamura et al. demonstrated the correlation between the species evolution, the lowering temperature of the paleoclimate and the fragmentation of habitat in the Cenozoic using the *Drosophila* group as a model for the analysis (Tamura et al. 2004). Russo et al. assumed utilisation of the variety of new fruits of flowering plants as one of the possible factors in the speciation in the *Drosophila* group, resulting in *Drosophila* specialisation and their ecological diversity (Russo et al. 2013). We concluded that similar patterns of speciation observed in grass flies due to the drying of the climate at the end of the Miocene and were related less to formation of grass host plants than to the increase in plant abundance. The adaptation of fly larvae to other species of host grasses, especially to the species of another Subtribe, could be a factor triggering a new speciation in the “*variegata*” cluster. The change of host plant was important in speciation of some dipteran from Tephritidae: *Eurostasolidarines* Fitch, 1855 (Craig et al. 1993, Stireman et al. 2005) and *Rhagoletispomonella* Walsh,1867 (Forbes et al. 2005). Based on our data and calculations, *M.bohemica* diverged from *M.rufa* earlier than 0.25 MA. *M.bohemica* feeds on grasses from subfamily Loliinae while *M.rufa* feeds on Aveninae. One can assume the earlier origin of *M.rufa* in evolutionary lineage *M.femorata* - *M.rufa* - *M.bohemica* due to the earlier origin of genus *Koeleria* Persoon, 1805 (about 7 MA) compared to *Loliumperenne* (2.5 MA). *M.laeta* and *M.variegata* are adapted to different grass species from genus *Festuca* L. 1753 and *Avena* L. 1753. *M.femorata* partly feeds on the same grasses as *M.variegata, M.laeta* and *M.mosquensis*, except for plants from the supertribe Triticeae.

The earliest by origin, Supertribe Triticodae includes *Elymus* sp. grasses. According to Tsvelev (Tsvelev 1987), an unusually wide variability of hybrid grass species, including *Elymusrepens* (L.) Gould, contributed to rapid evolution of the species. Development on evolutionarily more ancient and variable grass species suggests that grass flies should have morphological, physiological and behavioral features close to those of the ancestral species of the cluster, which were wide oligophages. Two oligophage species meet this criteria: *M.variegata* is close to ancestral haplotype and *M.mosquensis* represents a separate branch from the ancestral haplotype (Tables 1, 2, 3, Fig. 1).

From the results indicated above, a connection can be assumed between the early origin of plants and their species diversity and the distribution and formation of initial groups of species of grass flies. Climate change and variability of ecological conditions has resulted in the divergence of grass flies, as they adapt to evolutionarily younger, more abundant grasses with expanded distribution ranges. Herbert et al. demonstrated that the reproductive isolating barriers in *Phytomyzaglabricola* Kulp, 1968 (Diptera: Agromyzidae) were associated with different host plants (Herbert et al. 2013).

In general, preferences of grass fly feeding are independent from the ecological characteristics of grasses, except for *M.rufa* which feeds on firm bunchgrass, *M.laeta* - on short grasses and *M.variegata* - on tall and semi-tall grasses. Tall grasses (*Elymusrepens*), unlike short grasses, are characterised by high shoots, large and raw stems and leaves, as well as low stooling. *M.variegata* and *M.mosquensis* are more xerophilic, which may be more consistent with an early origin.

Various methods of statistical analysis demonstrated the set of the most informative differentiating traits of studied grass flies which allowed to differentiate the species with an accuracy of 95 - 99%. The highest classification accuracy is achieved when using PNN (99%) and the lowest when using LDA (95.8%). The accuracy of classification using RDA and FDA is 97.9% and 98.96%, respectively. The outcomes from different methods of analysis lead to the conclusion that the most important differentiating features of species in the “*variegata*" cluster are the traits S, h1 and Plant.

Input data in PNN analysis without Plant trait decreases the classification accuracy indicating the importance of the differentiating role of the trait Plant in this group of flies. Obviously, the contribution of the Plant trait is not limited only to the number of plant species because each species of grass flies in each evolutionary lineage of “*variegata*” cluster is associated to a specific host plant for its development (Table 3). It is an indication of importance of original plant species in the evolution of grass flies, besides the number of species of host plants.

We think that the area of the anterior process of the postgonite is the most significant criterion of species division. The trends of shape change of the anterior process are less visible than the change of its size (Table 1, Fig. 1B). The Mahalanobis distance calculation confirmed the uniformity of the shape of the anterior process of postgonite both between the “*variegata*” and “*inornata*” clusters and within the “*variegata*” cluster (*M.mosquensis – M.variegata, M.femorata – M.variegata*) (Yatsuk and Safonkin 2018). However, the non-overlapping dimensions of the anterior process indicate its important role in reproductive isolation.

Possibly, geographic isolation between the East Asian *M.inornata* and the species of the “*variegata*” cluster resulted in similarity in shape and size of the anterior process of the postgonite in *M.inornata* and *M.variegata* (Table 1).

The revealed trends in the evolution of the “*variegata*” cluster, based on the external key features, may be correlated with different adaptations of grass flies to the environment. Pale colour of mid-stripes and occipital spot, but bright occiput stripes (except for *M.mosquensis* and *M.elbergi*), may be due to adaptation of *Meromyza* flies to the colour range of the host plants. The considerable thickening of hind femurs may be an adaptation of *Meromyza* flies mainly to the jumping motion along the stems as a characteristic of the adult behaviour of the genus *Meromyza*. Black setae on the lower surface of gena are the characteristic of the West European origin of some species (Safonkin et al. 2018). Many features, similar to those of *M.inornata*, have been revealed for *M.mosquensis* and, to a lesser degree for *M.elbergi* (Table 1), suggesting that these features have been rooted in a common hypothetical ancestor of both clusters.

Lacking genetic data for *M.elbergi*, we can use only external features and the structure of the postgonite for reconstruction of this species phylogenetic position in the cluster (Fig. 1A). The first hypothesis is that *M.elbergi* could originate from the *M.femorata* “lineage” since it is close to *M.rufa, M.bohemica* by postgonite structure. The second is that *M.elbergi* could originate from the *M.mosquensis* lineage, since it has a combination of external key features inferred for the ancestral haplotypes of the clusters (*M.inornata* – *M.mosquensis*).

## Conclusions

This study highlights that the adaptation to different host plants could be the main factor in divergence of grass flies of the “*variegata*” cluster. We think that formation of the specific set of external features, which are rather uniform within a cluster, was associated with the developing of grass flies in similar conditions of the grass biome. Stabilising selection for a set of species external feature resulted in the formation of differences in structure and size, with insignificant change in shape and specific features of the male genital apparatus. The increased availability of host plants could be directly connected to the distribution, diversification and the adaptation of these grass flies to narrow oligophagy, but the development of these changes started later than the diversification in the Pooideae subfamily of grasses and the distribution of these grasses in biomes in the Middle and Late Miocene.

## Acknowledgements

Our thanks to Dr Michael Blackburn, Invasive insect biocontrol and behaviour laboratory, ARS, USDA, Beltsville, MD and Dr Yelena Golubeva, Cancer Genomics Laboratory, DCEG, LBR, NCI for reading and editing the manuscript.

The statistical analysis and its interpretation was performed with support from the Russian Science Foundation (Grant No. 21-14–00123).

## Figures and Tables

**Table 1.** The characteristics of *M.inornata* and the species of the "*variegata* " cluster.

**Features**	***M.inornata***	***M.variegata***	***M.laeta***	***M.mosquensis***	***M.femorata***	***M.rufa***	***M.bohemica***	***M.elbergi***
	N = 20	N = 20	N = 20	N = 20	N = 20	N = 3	N =20	N =1
Colour of palpi	sometimes brown in distal part	sometimes brown in distal part	black	sometimes brown in distal part	half black	light	sometimes brown in distal part	half black
Setae on the lower surface of gena	light	sometimes black	light	light	sometimes black	light	often black	sometimes black
Ratio of the gena height to the height of the 3rd antennal segment	1.04 ± 0.06	0.82 ± 0.03	0.84 ± 0.03	0.85 ± 0.02	0.97 ± 0.03	0.64 ± 0.03	0.77±0.03	1.0
Ratio of the height of ocellar triangle to the base of triangle	1.01 ± 0.02	0.94 ± 0.02	1.05 ± 0.03	0.92 ± 0.03	0.96 ± 0.03	1.05 ± 0.03	1.17±0.04	1.35
Ratio of the length of triangle to the length of head	0.77 ± 0.01	0.59 ± 0.01	0.66 ± 0.01	0.56 ± 0.01	0.66 ± 0.01	0.61 ± 0.02	0.66±0.01	0.68
Occipital spot	yes	no	no	yes	no	no	no	no
Occipital strips	no	no	no	yes	no	yes	yes	yes
Colour of mesonotum strips	black	brown	brown-black	black	reddish	yellow-brown	brown	brown-black
Ratio of the length of head to the length of mesonotum	0.68 ± 0.02	0.62 ± 0.02	0.63 ± 0.01	0.61 ± 0.01	0.62 ± 0.01	0.71 ± 0.03	0.64 ± 0.01	0.73
Mid-strip of mesonotum	strip reaches the scutellum	strip does not reach the scutellum	sometimes passes through the scutellum	passes through the scutellum	strip does not reach the scutellum	strip does not reach the scutellum	strip does not reach the scutellum	strip reaches the scutellum
Ratio of mesonotum to scutellum	3.00 ± 0.12	3.14 ± 0.05	3.27 ± 0.07	3.23 ± 0.07	3.17 ± 0.06	3.37 ± 0.32	3.19 ± 0.06	2.92
Ratio of the width of hind femurs to the width of hind tibia	3.52 ± 0.10	3.64 ± 0.09	3.31 ± 0.09	3.28 ± 0.09	4.32 ± 0.15	3.83 ± 0.20	3.37 ± 0.06	4.0
Length of the body without abdomen	1.76 ± 0.06	1.80 ± 0.04	1.43 ± 0.02	1.44 ± 0.03	1.87 ± 0.04	1.49 ± 0.02	1.76 ± 0.03	1.51
Area of anterior process of the postgonite, µm²	8440.9 ± 49.5	9010.2 ± 134.3	4512.6 ± 91.6	5507.9 ± 87.0	7228.7 ± 93.1	3053.8 ± 296.3	4365.0 ± 139.4	4625.08
	N = 21	N = 20	N = 4	N = 22	N = 23	N = 4	N = 9	N =1
Area of species	East Asia	Polyzonal (Europe)	Polyzonal (Europe)	Boreal (Euro-Siberian)	Polyzonal (Europe)	Polyzonal (Europe)	Polyzonal (Europe)	Polyzonal (Europe)
Biotope	riverine meadow	groves, banks, forest edge	riverine meadow	meadows	groves, banks, swamp meadow side, dry meadows	flood meadow	groves, lowland meadow, near the roads	swamp and forest meadows, gardens, urban habitat

N - number of specimens measured, Х ± SE.

**Table 2.** The divergence of *M.inornata* and the species of the "*variegata*" cluster.

Species	Ma	
	Max	Min
*M.inornata* − the "*variegata*" cluster	6.58	1.67
*M.mosquensis*	2.27	0.58
*M.femorata*	1.57	0.40
*M.bohemica*+*M.rufa*	0.58	0.15
*M.bohemica*	0.25	0.06
*M.laeta*	0.25	0.06

Based on the BEAST programme, from Safonkin et al. (2020a).

**Table 3.** The speciation of host plants of species of the «*variegata*» cluster.

Tribe	Ma	Subtribe	Ma	Species of grasses	Ma	Species of grass flies
Poeae	33.5†	Agrostidinae	22†	*Agrostiscapillaris* L.	8.8	*M.femorata*, *M.laeta*, *M.mosquensis*
		Aveninae		*Koeleriacristata* (L.) Pers.	7.2	*M.rufa*
				*Avenasativa* L.	9.1	*M.variegata*
		Poinae	27†	*Phleum* *pratense* L., *Ph.phleoides* (L.) H. Karst.	−	*M.rufa*, *M.variegata*
				*Alopecuruspratensis* L.	9.2	*M.mosquensis*, *M.variegata*
				*Poa* sp., *Poapratensis* L.	9.4	*M.mosquensis*
		Loliinae		*Festucaovina* L.	3.6	*M.mosquensis*
				*Festucarubra* L.	1.8-3.3	*M.femorata*, *M.laeta*, *M.mosquensis*
				*Festucapratensis* Huds.	−	*M.variegata*
				*Loliumperenne* L.	2.9	*M.bohemica*
		Dactylidinae	8	*Dactylisglomerata* L.	4.3	*M.femorata*, *M.variegata*
Triticeae		Hordeinae	16	*Elymusrepen*s (L.) Gould	8	*M.mosquensis*?, *M.variegata*
				*Elymushispidus* (Opiz) Melderis	8	*M.mosquensis*

The time of speciation of the grasses and the time of divergence of lineages (†) is given according to Pimentel et al. (2017), − - no data, ? - supposedly.

**Table 4.** Results of the stepwise selection algorithm in the LDA model, including eigenvectors, the relative contribution of each function to species differentiation and the coefficients of canonical correlations.

Discriminant function	Eigenvalue	Relative percentage	Canonical correlation
1	26.8	85.79	0.98
2	2.89	9.26	0.86
3	1.06	3.40	0.72
4	0.385	1.23	0.53
5	0.086	0.28	0.28
6	0.0124	0.04	0.11

**Table 5.** Characteristics of the statistical significance of the separation of seven species of flies within the selected LDA model.

Discriminant function	Wilks Lambda	*Chi-Squared*	DF	P-Value
1	0.003	515.8	36	<< 0.01
2	0.082	221.6	25	<< 0.01
3	0.318	101.3	16	<< 0.01
4	0.657	37.2	9	<< 0.01
5	0.91	8.3	4	0.08
6	0.988	1.1	1	0.3

**Table 6.** Classification table of species based on LDA method (percentage of cases correctly classified - 95.83%).

Species	Actual number of individuals	Results of classification						
		*M.bohemica*	*M.femorata*	*M.inornata*	*M.laeta*	*M.mosquensis*	*M.rufa*	*M.variegata*
*M.bohemica*	9	**9**	**0**	**0**	**0**	**0**	**0**	**0**
*M.femorata*	20	**0**	**20**	**0**	**0**	**0**	**0**	**0**
*M.inornata*	20	**0**	**0**	**20**	**0**	**0**	**0**	**0**
*M.laeta*	4	**1**	**0**	**0**	**2**	**1**	**0**	**0**
*M.mosquensis*	20	**1**	**0**	**0**	**0**	**19**	**0**	**0**
*M.rufa*	3	**0**	**0**	**0**	**0**	**0**	**3**	**0**
*M.variegata*	20	**0**	**1**	**0**	**0**	**0**	**0**	**19**
Correctly classified	92	9	20	20	2	19	3	19
Incorrectly classified	4	**2**	**1**	**0**	**0**	**1**	**0**	**0**

**Table 7.** Classification table of species, based on RDA method (percentage of cases correctly classified - 97.92%).

Species	Actual number of individuals	Results of classification						
		*M.bohemica*	*M.femora*	*M.inorna*	*M.laeta*	*M.mosquensis*	*M.rufa*	*M.variegata*
*M.bohemica*	9	9	0	0	0	0	0	0
*M.femorata*	20	0	20	0	0	0	0	0
*M.inornata*	20	0	0	20	0	0	0	0
*M.laeta*	4	0	0	0	3	1	0	0
*M.mosquensis*	20	0	0	0	0	20	0	0
*M.rufa*	3	0	0	0	0	0	3	0
*M.variegata*	20	0	1	0	0	0	0	19
Correctly classified	**94**	9	20	20	3	20	3	19
Incorrectly classified	**2**	0	**1**	**0**	**0**	**1**	0	0

**Table 8.** Classification table of species based on FDA method (percent of cases correctly classified - 98.96%

Species	Actual number of individuals	Results of classification						
		*M.bohemica*	*M.femorata*	*M.inornata*	*M.laeta*	*M.mosquensis*	*M.rufa*	*M.variegata*
*M.bohemica*	9	**9**	**0**	**0**	**0**	**0**	**0**	**0**
*M.femorata*	20	**0**	**20**	**0**	**0**	**0**	**0**	**0**
*M.inornata*	20	**0**	**0**	**20**	**0**	**0**	**0**	**0**
*M.laeta*	4	**0**	**0**	**0**	**4**	**0**	**0**	**0**
*M.mosquensis*	20	**0**	**0**	**0**	**0**	**20**	**0**	**0**
*M.rufa*	3	**0**	**0**	**0**	**0**	**0**	**3**	**0**
*M.variegata*	20	**0**	**1**	**0**	**0**	**0**	**0**	**19**
Correctly classified	**95**	9	20	20	3	20	3	19
Incorrectly classified	**1**	0	**1**	**0**	**0**	**0**	0	0
